# Open science for nutrition and physical activity research: a new challenge and lots of opportunities for IJBNPA

**DOI:** 10.1186/s12966-018-0739-4

**Published:** 2018-10-24

**Authors:** Russell Jago, Hidde van der Ploeg

**Affiliations:** 1 Centre for Exercise, Nutrition & Health Sciences, School for Policy Studies, University of Bristol, 8 Priory Road, Bristol, BS8 1TZ UK; 2 Department of Public and Occupational Health, Amsterdam Public Health Research Institute, Amsterdam UMC, Vrije Universiteit Amsterdam, van der Boechorststraat 7, 1081BT, Amsterdam, the Netherlands

## Abstract

Open science has received a great deal of attention across the academic community. Open science has been defined in many ways, but the central principle is to make methods, results and the underpinning data (publicly) available. The aim of this editorial is to outline the key issues that the nutrition and physical activity fields need to consider in relation to making the research within the field as transparent and reproducible as possible. We provide a brief overview of the key issues to consider and provide open science recommendations for publications within IJBNPA and the wider field.

Open science has received a great deal of attention across the academic community. Open science has been defined in many ways, but the central principle is to make methods, results and the underpinning data (publicly) available. Open science principles are receiving key support from national funding agencies [1]. Interest in ways to promote open-science is being fuelled by thoughtful discussions about research waste [2], research reproducibility [3] and concerns about hidden clinical trial data [4]. While some fields have pushed open-science and the promotion of research reproducibility [5, 6] there has been less discussion about these issues in relation to the field of behavioural nutrition and physical activity research. As current and former Editors of IJBNPA we think it would be of tremendous benefit for the field and the populations that our research seeks to serve if we increased the transparency of our research. The challenge is to identify the specific steps that we can take to advance these issues while also recognising the challenges along the way.

In terms of steps that can be taken now, it is a requirement for IJBNPA that all trials should be registered and although IJBNPA does not publish protocol papers there are many excellent journals within our publisher’s (BMC) portfolio such as Trials and Pilot and Feasibility Studies that accept protocol papers. We therefore strongly encourage publication of protocols in these journals or other appropriate venues. As outlined in the author guidelines, we also expect all authors to use the appropriate reporting checklists (CONSORT, STROBE and TIDIER [7]) for all trials and cohort papers. In terms of reviews, it is easy and free to register reviews on PROSPERO and there would seem to be no reason why this cannot be done for all IJBNPA reviews. We encourage all reviews to be registered on PROSPERO and this will become a formal requirement from January 2020.

Making data fully available to facilitate replication should be a core aspiration of our field. We recognise that there can be challenges in making data available and while this is a requirement for some funding agencies, it is not for all. However, there are many repositories and institutional portals that are simple to use, and we encourage all authors to make data available where possible. We also think that providing analysis code (syntax, script, do-file or whatever term you choose) for new techniques or methods along with detailed descriptions of what the code is trying to do would be a relatively easy goal for the field. While providing code for standard commands would be unnecessary, code for novel applications such as the creation of food groups from national dietary surveys or new means of processing raw accelerometer data would be of great help to the field. While we recognise that code often represents huge amounts of thought and work by statisticians that often do not get sufficient credit for their invaluable contributions, providing code as a supplementary file that is citable would provide the justifiable credit and allow the field to learn from each other, and make our research more efficient. We therefore encourage all authors to consider if code could be made available in supplementary files and we will actively promote data sharing for the journal.

It is important to recognise that there are other strategies that could be adopted by the field to facilitate open science such as the use of registered reports and pre-prints. Registered reports, in which publication decisions are taken on the methods and importance of the research question posed before data are collected have drawn interest from many fields including psychology and associated behavioural health fields such as addiction research [8] and could be part of the future for nutrition and physical activity research. A similar initiative that is currently tested within BMC is peer review blinded for the findings of completed studies, which would help reduce publication bias. The benefit of this method is that some indicators of study execution quality could still be made available to the reviewer, which would for example give the journal the opportunity to determine retention rates and external validity. Alternatively, we could look to push ahead with the use of pre-prints such as the service provided by bioRxiv in which manuscripts are made available prior to peer review, thereby facilitating rapid knowledge translation but without the external scrutiny that is the hallmark of peer review. Each of these approaches poses opportunities and threats both in practical terms and in the general understanding of the how nutrition and physical activity research is published. The challenge for the field is therefore to have a broader discussion about how best to facilitate open science principles within nutrition and physical activity research and the steps that we can take to increase the transparency and replicability of our work. We therefore think that as a field we should discuss the applicability of these approaches as well as others that may be available to identify the most effective means of making our research as open as possible.

In conclusion, we encourage all authors to consider the issues presented in this editorial, to adopt the steps that can be taken now and rapidly move to data and code sharing as means of advancing the fields of nutrition and physical activity research.
